# Intranasal vs. Device-Assisted Drug Delivery: Advantages and Limitations for the Delivery of Biopharmaceuticals to the CNS

**DOI:** 10.3390/pharmaceutics18040484

**Published:** 2026-04-14

**Authors:** Lisa Benedetta De Martini, Chiara Flora Valori, Martina Morrone, Liliana Brambilla, Daniela Rossi

**Affiliations:** 1 National Center for Oncological Hadrontherapy (CNAO), 27100 Pavia, Italy; lisa.demartini@cnao.it; 2 Istituti Clinici Scientifici Maugeri IRCCS, Laboratory for Research on Neurodegenerative Disorders, 27100 Pavia, Italy; chiaraflora.valori@icsmaugeri.it (C.F.V.); martina.morrone@icsmaugeri.it (M.M.); liliana.brambilla@icsmaugeri.it (L.B.)

**Keywords:** drug delivery, Nose-to-Brain administration, device-assisted delivery, therapy

## Abstract

While the Blood–Brain Barrier (BBB) is essential for the protection and function of the Central Nervous System (CNS), it also represents a challenge for drug delivery in the treatment of CNS disorders due to its limited permeability and high expression of efflux transporters. Crossing the BBB becomes even more difficult when dealing with biomolecular therapeutics (e.g., monoclonal antibodies and Antisense Oligonucleotides) due to their hydrophilic nature and high molecular weight. Over the years, different strategies have been developed in order to maximize the ability of biopharmaceuticals to cross the BBB and be delivered to the CNS. Both non-invasive techniques, mainly consisting of developing innovative vectors or using non-conventional routes of administration (e.g., intranasal delivery), and invasive methods, such as intracerebroventricular/intrathecal administration, have been tested individually and in combination. Given the improvements achieved nowadays with both approaches, here, we plan to compare the advances in invasive techniques, such as those based on the use of device-assisted strategies, and the employment of the intranasal route of administration. We are also interested in reporting the applicability of both strategies in the treatment of aggressive forms of cancer, such as glioblastoma, as well as neurodegenerative diseases, in order to determine which technique can be considered a better choice in each specific case.

## 1. Introduction

A primary challenge in the development of therapeutics for the Central Nervous System (CNS) is to secure the drug passage through the Blood–Brain Barrier (BBB), after systemic administration [1]. While ensuring the protection and the unimpaired function of the CNS, the BBB represents the main impediment to the delivery of systemically administered therapeutics to the nervous tissue due to the limited BBB permeability and the high expression of active efflux transporters. Access to the CNS is especially challenging when dealing with biopharmaceuticals, such as peptides, recombinant proteins, monoclonal antibodies and nucleic acids due to their higher molecular weight and hydrophilic nature [1,2]. Generally, a molecule should have both a molecular mass under 400–500 Da as well as lipophilic properties to cross the BBB via transcellular passive diffusion. Thus, this mechanism cannot be used by the totality of biopharmaceuticals and by 98% of small molecules [3]. Due to their structural complexity, biopharmaceutical drugs offer the advantage of exhibiting enhanced specificity towards the molecular target and higher potency compared to small molecules [4]. However, their composite structure makes them among the most challenging molecules to formulate and deliver. When dealing with biopharmaceuticals, three key aspects need to be taken into consideration: (i) the sensitivity to chemical and/or enzymatic degradation in physiological environments resulting in three-dimensional structure loss; (ii) the short plasma half-life linked to renal clearance; and (iii) the instability and membrane impermeability resulting in difficulty to reach the ultimate target [5,6]. Overall, these aspects hamper the efficient delivery of biodrugs to their target destination within the CNS [7]. For these reasons, local delivery strategies, such as intracerebroventricular, intraparenchymal convection-enhanced and intrathecal administration, which are able to directly introduce therapeutics to specific regions of the CNS bypassing the BBB, are still widely used to deliver biopharmaceuticals to the CNS, despite their limitations in terms of treatment allocation (e.g., need for hospitalization), safety, and costs [2,5]. In recent years, increasing research efforts have been made to develop technologies apt to convey therapeutics to the brain, without resorting to local delivery. Two main strategies have been proposed: (a) bypassing the BBB by using unconventional administration routes, such as the intranasal (IN) route, and (b) enhancing drug delivery through the BBB by means of formulation strategies or physical disruption [8,9]. Co-delivery with chemicals that affect BBB influx and efflux transporters; functionalization with brain-targeting ligands that result in nanoparticle-mediated delivery; and liposome- or viral-mediated delivery represent most of the formulation strategies used to ease biopharmaceuticals through the BBB [8,9]. On the other hand, the use of mechanic stimuli that modulate BBB permeability by causing its controlled physical disruption has also been explored. More specifically, the recent development of device-assisted strategies, such as Focused Ultrasound (FUS) and Electroporation, has resulted in promising invasive and non-invasive applications [8].

When developing therapeutic strategies for CNS disorders, there are also other important aspects that must be taken into account. For instance, a crucial difference that should be carefully considered is the one between chronic and acute diseases. Managing chronic disorders, such as Amyotrophic Lateral Sclerosis (ALS) or Parkinson’s Disease (PD), mostly requires the administration of a drug on a daily basis for a prolonged period of time. Therefore, patient compliance is essential for the treatment of these conditions. In these cases, the oral route of administration is the one with the highest level of acquiescence [10,11]. However, due to their sensitivity to pH changes, enzymatic degradation and membrane impermeability, biopharmaceuticals are often unsuitable for this route of administration [6]. Hence, other non-invasive and easy to administer routes may be explored for these types of medicaments, including IN delivery. Conversely, when dealing with an acute and/or aggressive CNS disorder, such as stroke or tumours, the main concern is the rapidity of action. The parenteral routes of administration are therefore considered more favourable. Overall, this amount of evidence suggests that different CNS disorders present diverse needs in terms of treatment strategies that must be carefully evaluated.

Given the recent advancements in the development of CNS delivery techniques, this review will provide (1) an overview of the BBB physiopathology in CNS disorders; (2) a comparison between the progresses in BBB disruption techniques, focusing on device-assisted strategies and the IN route of administration for the delivery of biopharmaceuticals into the CNS; and (3) an assessment of the efficacy of both approaches in the treatment of aggressive forms of cancer, such as glioblastoma multiforme (GBM), and neurodegenerative diseases.

## 2. Biological Barriers at the Blood-to-Brain Interface

A highly controlled microenvironment is essential to maintain the normal functioning of the CNS. This condition is preserved by the presence of a biological barrier at the blood-to-brain boundary, whose role is to physically separate the CNS from the rest of the body [9,12]. This biological barrier is composed of three key interfaces, which are characterized by different cell types: the BBB, which is mainly formed by microvascular Endothelial Cells (ECs); the Blood–Cerebrospinal Fluid Barrier (BCSFB), which is made of epithelial cells of the Choroid Plexus (CP); and the arachnoid barrier, which is constituted by the arachnoid epithelial cells surrounding the brain under the dura mater [12,13,14].

The BBB, localized between the bloodstream and the brain interstitial fluid, is the largest interface for blood–brain exchanges, with a combined surface area in an average adult of about 12–18 m^2^. It represents the main obstacle preventing the entry of blood-borne agents into the CNS, including drugs [15,16]. The BBB exerts its critical functions, owing to the interaction of tightly sealed brain ECs with mural cells (pericytes and vascular smooth muscle cells), astrocytes, neurons, microglia and a basement membrane. This highly regulated cell complex is referred to as the Neurovascular Unit (NVU), with every cell type playing a specific role [9,14,17]. Brain microvascular ECs possess unique morphological features, structural organization, and functional capabilities that distinguish them from cells of the peripheral vascular endothelium [18]. More specifically, they are characterized by the expression of both Tight Junction (TJ) and Adherens Junction (AJ) proteins. While TJs seal the paracellular space between adjacent endothelial cells, thereby restricting paracellular transport of water-soluble molecules between the blood and the brain, AJ proteins contribute to the stabilization of the endothelium. Furthermore, ECs present a very low rate of transcytosis, which limits the transcellular exchange of molecules. Therefore, ECs resort to the expression of dedicated active transporter proteins to regulate the exchange of essential molecules, while blocking the transit of undesired substances [17,18,19]. The passage of exogenous substances, including drugs, is further reduced by the presence of various efflux transporters (designated as multidrug resistance proteins) as well as by the expression in the brain endothelial cells of a variety of drug-metabolizing enzymes (e.g., cytochrome P450 enzymes), which result in the formation of a metabolic barrier [14,20].

The second barrier of the CNS is represented by the BCSFB, which is formed by the fenestrated endothelial cells of the CP, covering roughly 1.7 m^2^ of the surface area that separates the blood and the ventricular Cerebrospinal Fluid (CSF) [9,16]. The CP is an epithelio-endothelial convolute, characterized by the presence of epithelial cells producing the CSF [21,22]. The TJs located between the CSF-facing parts of the CP epithelial cells constitute the BCSFB, which, together with the BBB, is essential for the homeostatic regulation of the CNS microenvironment [22,23]. However, compared to the BBB, the BCSFB is leaky. Small and large molecules present in the blood, including drugs, can penetrate into the CSF, at a rate that shows inverse proportionality to their molecular weight [24]. Although drugs can enter the CSF, their penetration into the brain tissue remains limited due to the rapid efflux back into the bloodstream [9,24].

The last barrier of the CNS is represented by the meningeal arachnoid barrier, located between the bloodstream and subarachnoid CSF [16]. This third barrier is formed by the arachnoid epithelium underlying the dura mater. Given its avascular characteristics and limited surface area when compared to the other barriers, it seems to have a limited role in blood–brain substance exchange [12].

Under normal physiological conditions, these barriers sustain a tightly regulated homeostatic environment within the CNS, thereby ensuring optimal brain function and protecting neural tissue from significant variations in ion and nutrient levels [8]. However, neurological disorders can modify the structure of the BBB and BCSFB, contributing to the physiopathology of the disease itself. These modifications need to be taken into account when designing therapeutic molecules and their delivery systems [7].

### 2.1. BBB Abnormalities Linked to CNS Disorders

BBB impairment has been documented in several CNS disorders, including brain tumours, particularly GBM [25], and neurodegenerative diseases, such as ALS [13,26], Multiple Sclerosis (MS) [27], PD and Alzheimer’s Disease (AD) [27]. Pathological barrier disruption can range from minor, temporary alterations in BBB permeability to persistent barrier damage. Hereafter, we will review the morphological and physiological changes seen in GBM and in various neurodegenerative disorders.

#### 2.1.1. BBB Disruption in GBM

Glioblastoma is the most common and lethal malignant primary brain tumour in adults. It is characterized by a median survival of less than 2 years. The incidence of glioblastoma increases after the age of 40 and peaks around the age of 75–84. Current standard of care is represented by total surgical resection, followed by radiation therapy and concomitant administration of the chemotherapeutic agent temozolomide (TMZ) [28,29,30].

In primary brain tumours as well as in brain metastases, the BBB is disrupted, and it is referred to as the Blood–Tumour Barrier (BTB) [31]. The BTB is considered leakier than the normal BBB, as it presents disrupted pericyte distribution as well as compromised astrocytic endfeet and neuronal connectivity [31]. This breakdown is linked to neuroinflammation due to the production and release by GBM cells, pericytes and astrocytes of inflammatory mediators into the tumour microenvironment [32,33]. Increased permeability of the NVU is thought to also be caused by the infiltration of circulating immune cells into the CNS and the concomitant decreased expression of junctional proteins [31]. However, it has become clear that the BTB is heterogeneously permeable, as its integrity is altered in proximity of the GBM bulk, while it is preserved in the brain tissue-adjacent-to-tumour area, where isolated GBM cells are present [29,31]. Accordingly, recent single-cell transcriptomic and histopathological analyses of GBM biopsies revealed the distinct molecular fingerprint of three classes of ECs in the tumour core, characterized by the overexpression of genes associated with angiogenesis and vascular leakage. In addition, two other populations were identified in the tumour periphery and were defined by a quiescent phenotype [34].

The differential integrity of the BTB, together with the distinct expression of active efflux transporters, prevents therapeutic agents from reaching all GBM cells, especially those located in the brain-adjacent-to-tumour area. This can cause therapy failure and relapse [27,29,31]. Difficulties in accessing each and every GBM cell underline the need for both novel therapeutic agents and delivery systems in order to enhance the conveyance and retention of therapeutics within the tumour microenvironment [8,30,35].

#### 2.1.2. BBB Changes in Neurodegenerative Disorders

Evidence of endothelial degeneration and impaired BBB function has been reported in different neurodegenerative diseases, such as ALS, MS, PD and AD. In these scenarios, BBB breakdown was reported to cause ion dysregulation, oedema and neuroinflammation, ultimately resulting in neuronal dysfunction [36]. Moreover, emerging research indicates that morphological and molecular modifications also in the BCSFB are implicated in the underlying physiopathology and progression of neurodegenerative disorders. Hereafter, we will describe the most significant BBB and BCSFB alterations identified in distinct neurodegenerative disorders.

ALS is a fatal neurodegenerative disease, resulting in progressive muscle weakness, ultimately leading to paralysis and respiratory complications [25,37]. Treatment options for ALS are limited and mostly focused on improving quality of life [37].

Alterations of the BBB have been reported in ALS, including morphological modifications of ECs, astrocytes and pericytes; changes in the expression of TJ and AJ proteins; and increased permeability [25]. In particular, degeneration of ECs and pericytes was reported to be accompanied by diminished capillary pericyte coverage and activation of astrocytes and microglia, thereby emphasizing the presence of a neuroinflammatory state. Infiltration of circulating erythrocytes and immune cells, and also blood-derived molecules—e.g., immunoglobulins G, albumin, thrombin, or fibrin—has been observed, in accordance with the reduction in TJ and AJ proteins promoting paracellular permeability [13,25]. Some changes have also been reported for the BCSFB, although little is known about the role of the CP in ALS physiopathology [23].

MS is an autoimmune disease affecting the CNS and characterized by inflammation, demyelination, gliosis, and neuronal loss [38]. MS exhibits considerable clinical heterogeneity, with different degrees of severity, from initial Clinically Isolated Syndrome (CIS) to the predominant subtype Relapsing–Remitting form (RRMS), which may switch to Secondary Progressive MS (SPMS), characterized by permanent neurological deficits and disability. Alternatively, Primary Progressive MS (PPMS) may present as a progressive deterioration in a subset of patients starting from the disease onset [39,40]. Currently, several Disease-Modifying Treatments (DMTs) have been approved by the European Medicines Agency (EMA) for the treatment of RRMS. However, the progressive forms of MS pose significant challenges, given the small number of available therapies with proven efficacy [38,40,41].

BBB disruption is a central feature of MS, and it is related to the presence of a neuroinflammatory environment. More specifically, deregulation of TJ and AJ proteins occurs concomitantly with perivascular astrogliosis, and it is linked to increased BBB leakiness and immune cell infiltration [26]. These BBB alterations are believed to occur during the initial stages of the lesion development, and they facilitate CNS immune infiltration through the brain barriers, a pivotal step in MS physiopathology [23,26]. MS does, in fact, feature multiple white matter inflammatory lesions accompanied by increased T-cell, B-cell, and macrophage infiltration into the CNS [23]. Dynamic contrast-enhanced Magnetic Resonance Imaging (MRI) studies showed that barrier leakage is almost always present in new lesions, while it is rarely observed in older ones [26,36]. Thus, it is reasonable to postulate that BBB disruption is a transient event. Even though the CP is thought to be an important entry site for lymphocytes into the CSF, damage of the BCSFB in MS has been poorly explored [23,26,42].

AD represents the most frequently diagnosed form of age-related dementia. This condition is progressive and ultimately fatal. It exhibits a multifactorial aetiology, with neuroinflammation being a key contributing factor [43]. AD pathology arises from structural and functional CNS damage. More specifically, it is characterized by aberrant protein aggregation (amyloid β plaques and tau tangles) and progressive neurodegeneration [44]. Therapeutic approaches for AD, which are mostly based upon anticholinesterase inhibitors (donepezil, galantamine, and rivastigmine) and anti-glutamatergic compounds (memantine), are focused on symptom management rather than disease modification [44,45]. DMTs were made available on the market only in recent years. These consist of the monoclonal antibodies Aducanumab and Lecanemab, which target amyloid β (Aβ) plaques [46,47].

BBB and BCSFB alterations have been observed in AD. BBB disruption is linked to the neuroinflammatory process triggered by the amyloid β-induced release of cytokines. It manifests with increased permeability of serum proteins into the parenchyma, irregular basement membrane and changes in EC protein expression [48]. Distinctive structural changes have also been reported in BCSFB of AD patients, including atrophy of the epithelial cells, as well as thickening of the basal membrane and vessel walls [49]. Moreover, accumulation of intracellular inclusions (i.e., Biondi ring tangles) and presence of amyloid β deposits has been spotted in CP epithelial cells [50]. These are thought to be associated with immune responses leading to capillary damage and fibrosis [23]. Lastly, a larger CP volume, as assessed by MRI, has been correlated with severity of cognitive impairment in AD patients [51].

PD is the fastest growing neurological disorder in terms of age-standardized rates of prevalence, disability, and death [52]. It is commonly known for the deregulation of the dopaminergic system due to loss of dopaminergic neurons in the substantia nigra pars compacta. PD is actually a heterogeneous syndrome, with each patient presenting with a distinct set of symptoms [53]. A characterizing aspect of PD is the presence of abnormal intracellular aggregates of α-synuclein, referred to as Lewy bodies [54]. Currently, no DMTs are available for PD, leaving it with an unmet medical need [55].

Although early research on BBB permeability in PD showed no obvious disruption, in more recent studies, extravasation of serum proteins and red blood cells was detected in the CNS, underlying increased permeability of the BBB [56]. Furthermore, in post-mortem brain tissue from PD patients, increased BBB permeability was found in conjunction with altered TJ protein expression, widely spread neuroinflammatory responses by microglia and astroglia, and inflammation-related pericyte phenotypic changes. To date, it has not been yet elucidated whether BBB disruption and α-synuclein deposits are the cause or the result of neuroinflammation [56]. Also, little is known about the role of BCSFB in the regulation of α-synuclein in the CSF as well as in PD pathogenesis [23].

#### 2.1.3. Relying on BBB Pathology-Dependent Disruption for Drug Delivery

While investigating pathology-related changes in the BBB remains an important issue to address, it has become increasingly evident that it is not possible to rely only on disease-related BBB leakiness to deliver drugs to the CNS [7]. Disease progression may, indeed, influence both the magnitude and the duration of the BBB opening. For instance, as previously mentioned, BBB disruption in MS has been associated with the early stages of lesion development, while it is absent in late lesions [36]. Another effective example is represented by tumours, where changes in BBB permeability are dependent on both the type of tumour and the size of the lesion. The endothelial barrier remains, indeed, largely intact in low-grade astrocytomas but becomes significantly disrupted in high-grade astrocytomas, glioblastomas, and metastatic brain tumours [57]. Moreover, as previously reported, BBB opening in GBM is heterogeneous, with areas of increased permeability only in proximity of the GBM bulk. This implies that if the delivery strategy is meant to exploit disease-related openings, the cells in the brain-adjacent-to-tumour area would not be affected. This represents the main problem with TMZ resistance [30]. The development of novel strategies is thus essential to avoid local delivery, which exposes the patients to a greater risk of brain damage due to its invasive nature [58]. Hereafter, we will review different dispensing strategies, and we will focus on those with possible non-invasive applications, including device-assisted delivery.

## 3. Device-Assisted Drug Delivery

Device-assisted drug delivery systems are technological platforms that use mechanical, electronic, or other engineered devices to control, enhance, or facilitate the delivery of therapeutic agents to the target. The application of device-based technologies has been proposed as a strategy for systemically administered therapeutics to overcome the BBB and to access the CNS. Current device-based approaches enhance BBB permeability either by barrier disruption or complete BBB bypass. Techniques based upon the disruption of the BBB include Magnetic Resonance-Guided Focused Ultrasound (MRgFUS), Laser Interstitial Thermal Therapy (LITT), Transcranial Magnetic Stimulation (TMS), and electroporation, whereas BBB-circumventing methods comprise Convection-Enhanced Delivery (CED) and sustained delivery through implantable devices [8]. Among the aforementioned device-assisted delivery strategies, non-invasive methods have been developed and are currently under investigation only for MRgFUS, electroporation and TMS. In fact, LITT, CED and sustained delivery strategies rely on the insertion of a probe, catheter or implant directly into the brain parenchyma [8,59]. For the sake of this review, we will focus on techniques with non-invasive applications that aim to regulate BBB opening. These approaches will be compared with the IN route of administration, a non-invasive technique that aims at delivering drugs to the CNS by bypassing the BBB.

### 3.1. Magnetic Resonance-Guided Focused Ultrasound (MRgFUS)

The first scientific evidence of ultrasound-mediated effects on living biological tissues was documented in 1927. From that moment on, the interest in therapeutic applications of FUS significantly grew, principally because of the ability of high-intensity ultrasounds to create targeted lesions in the brain by inducing thermal necrosis for the treatment of neurological disorders [60,61,62].

The real breakthrough in the use of this technique came with the combination of FUS with an imaging technique, i.e., MRI, which allowed for the visualization of the anatomical structures in the target and provided continuous temperature mapping for real-time therapy control [60,63]. The integration of FUS technology with MRI guidance culminated in the development of MRgFUS. This technique alone resulted in a thermoablation system approved by the Food and Drug Administration (FDA) for essential tremor and tremor-predominant PD [60,64,65,66].

MRgFUS technology is gaining recognition for its capacity to deliver precise, non-invasive, and targeted therapeutic ultrasound energy, which can be modulated to generate diverse beneficial biological effects for neurological interventions [67]. MRgFUS applications include thermoablation, radiosensitization, immunotherapy, BBB opening for CNS therapeutic delivery, and neuromodulation [60,67]. However, a primary challenge in FUS development is maximizing targeting accuracy, while minimizing procedural complexity. For example, the skull can cause significant beam distortion due to the speed-of-sound discrepancy between bone and brain tissue, combined with severe ultrasound wave attenuation through the skull bone. Moreover, the trabecular layer of the skull induces heterogeneities in both speed of sound and density that lead to strong phase aberrations of the acoustic beam. In particular, higher frequencies increase the defocusing effects of the skull as the wavelength reaches the trabecular dimensions (~1 mm), while lower frequencies enlarge the focal region and increase the risk of inertial cavitation-induced tissue damage [68]. Low-intensity ultrasounds show minimal thermal effects, while they exert mechanical activities that can contribute to increased transient BBB permeability. In non-human primates, intermediate frequencies around 500 kHz—with a 3 mm wavelength—have been shown to achieve safe, reversible BBB opening, balancing targeting precision and safety without requiring phase aberration correction. This enables enhanced delivery to the CNS of various therapeutic agents, including hydrophilic molecules and biodrugs [67,68,69,70,71]. Although the mechanical effects of MRgFUS pose less risks than the high-intensity ultrasound thermal effects, excessive mechanical force may result in capillary disruption and erythrocyte leakage [72].

Given the crucial role of the BBB in maintaining CNS homeostasis, it is clear that its widespread and unselective disruption may result in parenchymal exposure to pathogenic agents, osmotic alterations, and xenobiotic substances, leading to potential neurotoxic effects. Therefore, it is imperative for any BBB opening strategy to be tunable with fine spatial and temporal precision. In rodents, non-human primates as well as humans, MRgFUS was reported to determine highly regulated and reversible BBB disruption, owing to the precise modulation of acoustic energy deposition. In non-human primate studies, the observed targeting error was sufficiently small (2.5 ± 1.2 mm laterally, 1.5 ± 1.3 mm along the depth axis, and 3.1 ± 1.3 mm in total) to enable the specific targeting of substructures of the basal ganglia, such as the associative or oculomotor caudate. Consistently, no serious clinical or radiological side effects were described in humans [62,68,73,74,75]. Furthermore, better control of MRgFUS-induced BBB opening could be achieved by the concomitant intravenous (IV) injection of microbubbles. These are defined as gas-filled polymeric microspheres that exhibit oscillatory behaviour characterized by periodic expansion and contraction when exposed to ultrasonic fields. This implementation enabled a decrease in the ultrasonic energy required for BBB permeabilization, thus diminishing brain damage [8,74,76,77,78]. Consistently, conventional approaches for BBB opening are based on the use of microbubbles as cavitation nuclei to enhance the effects of ultrasounds, together with the co-administration of therapeutic agents. Yet, microbubbles were also proven to be an efficient delivery agent when loaded with therapeutics and were shown to be capable of releasing them into the targeted area upon ultrasound application [72,74,76,78,79].

#### MRgFUS-Aided Drug Delivery of Biotherapeutics to the CNS

As previously stated, the main obstacle to the entry of biopharmaceuticals into the CNS is linked to their hydrophilic nature and high molecular mass. Yet, MRgFUS was shown to mediate safe disruption of the BBB, thus enabling the selective delivery of hydrophilic compounds to the nervous tissue. In particular, Airan et al. [70] demonstrated the effective delivery into predetermined brain tissue targets in rats of two hydrophilic molecular imaging agents, targeting glutamate carboxypeptidase II (GCPII), by using a microbubble-enhanced MRgFUS technique.

Furthermore, transcranial pulsed FUS, combined with microbubbles, was shown to enhance, in mice, the CNS delivery of recombinant human alpha-L-iduronidase (83 kDa), an enzyme that is deficient in mucopolysaccharidosis type I. This paved the way for a possible replacement therapy for the treatment of this neurological condition [80]. Efficient CNS delivery of a recombinant protein, through the MRgFUS technique, was also achieved in humans with the successful delivery of recombinant glucocerebrosidase (GCase, 55 kDa) for the treatment of a genetic form of PD. Specifically, Meng et al. [81] demonstrated, in the open label phase I trial NCT04370665, that three bi-weekly non-invasive MRgFUS GCase treatments at escalating doses could determine targeted delivery to the putamen, with a high degree of anatomic specificity, in the absence of severe adverse events.

Promising results have also been achieved with the delivery of monoclonal antibodies (mAbs) for the treatment of brain tumours. For example, studies in mice have proven that non-invasive MRgFUS is effective in delivering Trastuzumab to the CNS, a humanized mAb used for the treatment of breast cancer [76]. Kinoshita et al. showed that CNS site-specific local delivery of mAbs can also be achieved with the ultrasound and microbubble system in vivo with acceptable histological damage due to BBB disruption, when compared to catheter insertion into the brain [76,77].

In mouse models, safety and efficacy of in vivo CNS delivery of mAbs through MRgFUS was later confirmed for the human mAb Aducanumab (approximately 150 kDa), which targets Aβ [82,83]. In particular, Kong et al. [82] demonstrated that MRgFUS with microbubbles significantly improves CNS delivery of systemically administered Aducanumab through a transient hippocampus-localized BBB opening, lasting a maximum of 24 h. These results were recently confirmed by Jeong et al. [83], who also demonstrated a correlation between the efficiency of Aducanumab delivery in targeted areas and the extent of MRgFUS-induced BBB opening, suggesting that MRgFUS may be a valuable procedure to reduce the required infusion doses.

Of note, MRgFUS has recently seen its first human application for the delivery of mAbs into the CNS [84,85]. In particular, a prospective, single-arm, non-randomized study (NCT03714243) was performed to evaluate the safety and feasibility of MRgFUS as a tool to deliver Trastuzumab to brain metastases of Her-2-positive breast cancer. This trial provided first-in-human evidence of non-invasive and spatially targeted mAb delivery to the CNS, showing that MRgFUS was able to enhance the delivery of Trastuzumab to CNS lesions after intravenous administration. In patients, these lesions displayed increased uptake of the mAb in the absence of serious adverse events (SAEs) [84]. MRgFUS coupled with therapeutic-loaded microbubbles, referred to as microbubble-drug conjugates (MDCs), has also been investigated for the CNS delivery of nucleic acids for gene therapy [79,86]. Huang et al. [86] showed how plasmid-loaded microbubbles paired with non-invasive MRgFUS can determine successful delivery of plasmid DNA to the CNS, specifically into the cytoplasm of neurons localized in the sonicated area by enhancing endocytosis.

In addition, MRgFUS was coupled with MDCs covalently conjugated to antibodies with binding specificity for adeno-associated viruses (AAVs) to enhance the efficiency of viral vector-mediated gene therapies in the CNS [79]. Recombinant AAVs have been proven to be a successful delivery system, able to be taken up by their target cells, where they reach the nucleus and deliver their DNA cargo [87]. Trinh et al. [79] demonstrated in vivo the efficacy of the combination of MRgFUS with AAV-conjugated microbubbles in achieving the expression of a therapeutic protein in targeted areas of the brain, particularly the striatum and the substantia nigra.

The MRgFUS technique does not only support MDCs as a drug delivery system, but its applications in conjunction with infusion of nanoparticles have been investigated. In particular, gold nanoparticles have emerged as promising drug carriers in nanomedicine due to their ability in shielding, modulating half-life and promoting drug delivery to the target tissue, while maintaining their inert and non-toxic characteristics [88,89,90]. On the other hand, gold nanoparticles exhibit limited permeability across the BBB when administered independently, thereby requiring investigations on combined therapeutic approaches exploiting MRgFUS [90]. Etame et al. [91] proved the ability of MRgFUS to enhance the penetration of 50 nm PEGylated gold nanoparticles across the BBB into brain tissues, limiting their uptake into the liver and the spleen, where they would tend to accumulate.

MRgFUS application in cell therapy has also been assessed as a way to reduce the risks associated with invasive intracranial surgery, i.e., the procedure currently needed to implant cells into the brain parenchyma. Burgess et al. [92] demonstrated the suitability of employing MRgFUS to deliver neural stem cells to targeted areas of the rat brain, while preserving their undifferentiated state and potential to differentiate into neurons.

In conclusion, MRgFUS has enabled researchers to achieve promising results in the targeted delivery of different biodrugs to the CNS, paving the way to address increasingly complex medical challenges and unmet clinical needs.

**Figure 1:**
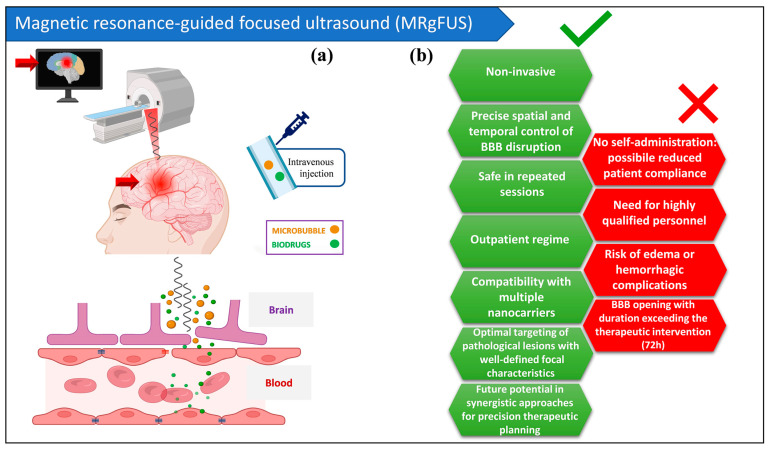
**Magnetic Resonance-Guided Focused Ultrasound.** (**a**) Representation of transiently disrupted BBB mediated by MRgFUS; this non-invasive technique, combined with intravenous injection of microbubbles, significantly improves CNS targeting of systemically administered biodrugs. (**b**) This panel shows the main strengths and limitations of the technique. Created in BioRender. Morrone, M. (2026) https://BioRender.com/v64stli (accessed on 30 March 2026) and modified using PowerPoint.

A comprehensive comparison of MRgFUS with other device-assisted BBB opening techniques, including assessments of key parameters—such as opening duration, spatial precision, clinical readiness, patient burden and technical challenges—is presented in Table 1. In paragraph 5, we will analyse the possible role of MRgFUS as a therapeutic strategy for different neurological disorders, while we represent the strengths and limitations of this technique in Figure 1.

### 3.2. Electroporation and Electric Field-Based Approaches

The second device-assisted drug delivery technology, with possible non-invasive applications, we will examine is electroporation. At variance with MRgFUS, electroporation uses Pulsed Electric Fields (PEFs) to induce membrane openings. Similar to FUS, it is suitable for a variety of purposes, from tissue ablation to reversible electroporation for drug delivery [93,94].

Electroporation was initially conceived as an in vitro technique for facilitating molecular transduction across cellular membranes. It can be described as a biophysical process by which application of external electric fields induces transient perturbation of the cellular membrane integrity, resulting in the formation of nanoscale pores within the lipid bilayer, which allow for intracellular delivery of different substrates [95].

Systematic study of electroporation at the cellular level begun in the 1980s, and the technique reached a breakthrough in 1982, when Neumann et al. demonstrated its suitability for mediating the effective transfection of plasmid DNA into mouse lyoma cells [96,97,98]. As a result, electroporation became one of the most popular non-viral cell transfection methods for both in vitro and in vivo applications [96].

Electroporation is a threshold-type phenomenon, meaning that different cellular biophysical responses can be triggered depending on the PEF parameters used, such as pulse duration, strength and frequency [94,99]. Upon PEF modulation, two main effects can be distinguished: (A) permanent permeabilization of the cell membrane, referred to as Irreversible Electroporation (IRE), which is caused by high intensity PEFs (>500 V/cm) and leads to cell death and tissue ablation, and (B) temporary permeabilization, referred to as Reversible Electroporation (RE), which is determined by low PEF strengths (100–500 V/cm), and it is characterized by transient pore formation, followed by membrane integrity restoration [94,95,99,100].

Similar to high-intensity focused ultrasound, IRE showed the capacity to induce tissue ablation. However, unlike ultrasound-based approaches, IRE operates through non-thermal mechanisms, thus allowing for the preservation of the structural and functional integrity of sensitive structures, including blood vessels and nerves adjacent to the treatment area [101,102,103]. The tissue ablation capabilities of this technique have made it of particular interest for the treatment of malignancies in different anatomical sites, such as the liver, kidney, lungs and CNS [104,105,106].

Recent technological advancements have emerged through modifications of pulse frequency and waveform characteristics. The transition to higher frequency square wave protocols led to the development of High-Frequency Irreversible Electroporation (H-FIRE). H-FIRE produced ablative lesions in rat brain tissue that were characteristic in cellular morphology of non-thermal IRE treatments. Specifically, there was complete uniformity of tissue death within targeted areas in the absence of muscle contractions, thereby showing the potential for clinical application without the need of paralytic agents [99,101,107]. Conversely, the shift towards sinusoidal waveform is the basis of the Burst Sine Wave Electroporation (B-SWE) method, which is characterized by diminished ablative capacity but presents increased versatility in terms of electrophysical outcomes [99].

In addition to the development of a non-thermal lesion, both H-FIRE and B-SWE induce BBB disruption [93,99,108,109]. In particular, H-FIRE was shown to transiently permeate the BBB up to 72 h after stimulation via cytoskeletal-mediated TJ modulation, thus determining a zone of transcellular BBB disruption that extends centimetres beyond the targeted area [108,109,110]. A recent study compared the effects in BBB disruption of H-FIRE and B-SWE, demonstrating that B-SWE exhibits a superior ability to facilitate BBB permeation into target tissues of large molecules, such as Evans blue dye (69 kDa). At the same time, it shows an accelerated restoration of the barrier function within 24 h post-treatment when compared to H-FIRE-induced BBB disruption [99]. Both techniques may be suitable for combination therapies, in which tissue ablation is coupled to the administration of chemotherapeutic or immunotherapeutic drugs, leveraging their capability to induce BBB disruption and enhance drug delivery [93,97]. It is, however, worth mentioning that both H-FIRE and B-SWE require invasive electrode placement directly within the CNS tissue, thereby exposing patients to greater risks of haemorrhage or infection once the approach will be transitioned to clinical settings [111].

Various electrode designs have been developed over time for IRE or RE, even though a standardized configuration does not yet exist due to the need for application-specific customization [94]. Electrodes are typically designed by taking into account the location of the target tissues, with invasive electrodes used for deep-seated targets and minimally invasive or non-invasive electrodes employed for superficial targets, such as exophytic tumours or melanoma [112,113].

In particular, non-invasive electrodes coupled with RE have seen applications in the treatment of cutaneous tumours, together with the Electrochemotherapy (ECT) method. The ECT methodology is based upon the capability of low-intensity PEFs, associated with RE, to increase cell membrane permeability, thus determining intracellular delivery of hydrophilic molecules [97,114,115]. The clinical application of ECT for cutaneous tumour treatment has become well established in medical practice [114]. The chemotherapeutic agents that are mostly used for ECT are bleomycin and cisplatin, which exhibit an objective treatment response rate of about 80% through this approach [114,116,117,118].

#### Low-Intensity Pulsed Electrical Fields and BBB Permeability

Consistent with the results achieved with IRE on BBB disruption, low-intensity PEFs (L-PEFs), applied using non-invasive electrodes pressed against both sides of the intact skull, have recently proved to increase BBB permeability in mouse models [119,120]. The application of L-PEFs, in the order of 10–100 V/cm, below the threshold for RE, was initially tested in vitro on a human BBB model. A significant increase in BBB permeability was found at voltages as low as 10 V by Sharabi et al. [121]. The same group then proceeded with a feasibility study in vivo on murine models, using L-PEFs ranging between 62.4 and 187.2 V/cm. Interestingly, they demonstrated that BBB disruption can be achieved with non-invasive protocols. Furthermore, L-PEFs were shown to be functional in determining increased permeability of the BBB for the passage of both small molecules, such as contrast agents (Gd-DOTA, 753.9 Da), and macromolecules, such as albumin bound to Evans blue (66 kDa) [119].

L-PEFs were later tested for the delivery to the CNS of the antineoplastic agent doxorubicin, a target of the efflux pump P-glycoprotein [120]. All mice treated with L-PEFs showed extended areas of BBB disruption with enhanced cortical penetration of intravenously administered doxorubicin at therapeutically relevant concentrations [120]. BBB permeabilization and doxorubicin concentrations exhibited spatial heterogeneity throughout the cortex, reflecting the uneven spatial distribution of the applied electric fields. The extent of barrier permeabilization seems to encompass a more extensive area than that observed with alternative methodologies, such as MRgFUS [120]. As yet, electroporation and electric field-based approaches have attracted less clinical attention than MRgFUS, with only two clinical trials submitted for GBM treatment (NCT03491683 and NCT05743595). However, in these cases, RE is used as a way to increase intramuscular absorption of biodrugs through the application of minimally invasive electrodes at the injection site, rather than to increase BBB permeability [122,123,124]. Conversely, L-PEFs demonstrate considerable clinical potential, particularly due to the non-invasive characteristics of this approach, even though further studies are needed to define the spatial and temporal extent of BBB disruption they can cause.

**Figure 2:**
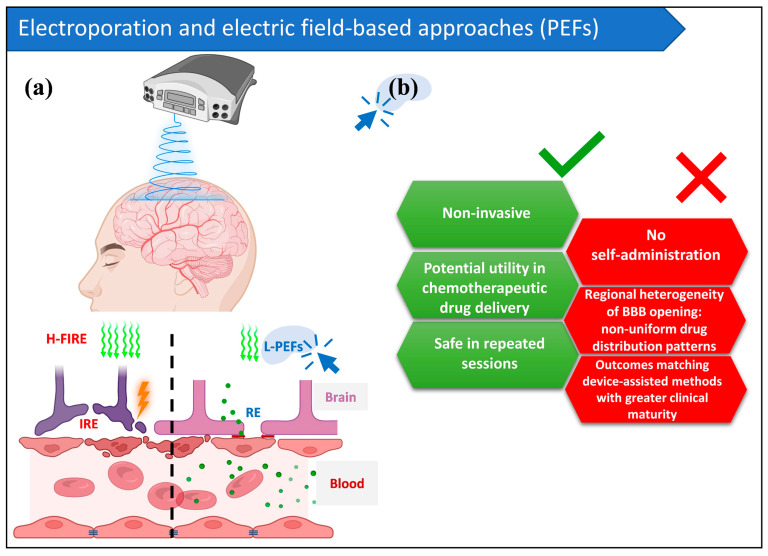
**Electroporation and electric field-based approaches.** (**a**) Schematic representation of Irreversible Electroporation (IRE) caused by high-intensity PEFs (H-FIRE) (*bottom, left*); Reversible Electroporation (RE) determined by low-intensity PEFs (L-PEFs) (*bottom, right*). The latter is a non-invasive technique that induces subtle, yet significant, BBB disruption, thereby increasing the permeability of the BBB to diverse molecules. (**b**) This panel shows the main strengths and limitations of L-PEFs. Created in BioRender. Morrone, M. (2026) https://BioRender.com/xqsxup3 (accessed on 30 March 2026) and modified using PowerPoint.

To date, L-PEF technology has not progressed beyond prototype development; no devices have been designed according to Medical Device Regulation standards, reflecting its preclinical status. For a detailed quantitative comparison of L-PEFs with alternative BBB disruption technologies across critical clinical and technical parameters, see Table 1. The strengths and limitations of L-PEFs as a CNS delivery technique are summarized in Figure 2.

### 3.3. Transcranial Magnetic Stimulation (TMS)

TMS is a non-invasive neurostimulation technique first developed by Barker et al. in the 1980s and later extensively investigated as a non-pharmacological therapeutic intervention for a broad spectrum of cognitive, psychiatric and neurodegenerative disorders [125,126,127,128]. TMS involves the application of short magnetic field pulses (50–500 μs) to the skull through the use of a wire coil that generates a localized magnetic field when positioned over the scalp [129,130]. TMS has three primary stimulation modalities: single-pulse TMS, paired-pulse TMS, and repetitive TMS (rTMS). While single-pulse and paired-pulse TMS may be used to assess brain functioning, rTMS is characterized by a pulse frequency of 1–50 Hz and was shown to induce changes in brain activity [129,131].

The pulsed magnetic field generated in rTMS exploits Faraday’s law of electromagnetic induction to produce long-term changes in synaptic strength with excitatory or inhibitory effects, depending on the frequency of stimulus [129]. Specifically, the electric fields generated by rTMS within neural tissue induce neuronal depolarization when applied at optimal intensity and orientation conditions [130]. Depolarization subsequently triggers glutamate release into the extracellular space, which correlates with transient BBB permeabilization through the activation of NMDA receptors [132,133].

Low-frequency (1 Hz) high-amplitude rTMS was, indeed, shown to safely and transiently increase BBB permeability in vivo in two different rodent models [132,134].

In vivo studies proved the efficacy of this technique in enhancing BBB permeability for both non-permeable small molecules, such as sodium fluorescein (NaFlu, 376.3 Da [135]), as well as macromolecules, such as the cytostatic biodrug insulin-like growth factor trap (IGF-Trap, ~400 KDa) [130,132]. The temporal dynamics of rTMS-induced BBB permeabilization have been characterized in rodents. Rapid BBB opening and subsequent closure, following rTMS application, was found, with an effective therapeutic window of less than 30 min, during which enhanced brain drug delivery was achieved [130]. This suggests that successful CNS drug delivery can be realized through this approach only when the pharmaceutical agent is administered concurrently to rTMS onset. Specific routes of administration, such as intravenous injections, should be then used in order to facilitate the rapid achievement of peak plasma concentrations [130]. This temporal constraint is not necessarily negative, provided that therapeutically effective drug concentrations are achieved within the CNS during this brief time window. The positive aspect is that such limited barrier permeabilization may reduce the risk of undesirable substance translocation across the BBB. Recent investigations have demonstrated that the combination of rTMS with systemic IGF-Trap administration produces therapeutic effects across two different animal models of glioma (murine and rat models). This suggests that rTMS may be beneficial in determining a sufficient BBB opening to achieve relevant drug concentrations in the brain [134]. The response to the combination protocol was however variable, being characterized by transient efficacy, followed by the development of treatment resistance [134]. These results highlight the need for further optimization of this technique and emphasize the requirement of evaluating diverse therapeutic agents to clarify its capacity to achieve adequate drug delivery concentrations to obtain effective treatments.

A positive note is that rTMS was proven safe in animal models when administered in repetitive sessions, over consecutive days. This suggests that recurring applications could be tested to improve effective drug delivery doses to the CNS in the absence of detrimental effects [130]. The first pilot trial in humans (NCT02474966) was also conducted to assess the safety of this technique. This trial confirmed that rTMS can safely and transiently increase BBB permeability to enable delivery to the CNS of systemic fluorescent and gadolinium-based tracers in patients with glioma [132].

As yet, several studies have been conducted with rTMS as a non-pharmacological therapeutic approach for a broad spectrum of neurological diseases. However, little is known about the true potential of this technique in enhancing drug delivery to the CNS. While recent advances in rTMS device design have enhanced pulse control and precision, increased complexity raised costs and maintenance demands that may limit widespread clinical applications, particularly in resource-constrained settings [136]. Nevertheless, the aforementioned study outcomes demonstrate considerable promise, particularly in light of the completely non-invasive nature of this approach and its potential for repeated therapeutic applications.

**Figure 3:**
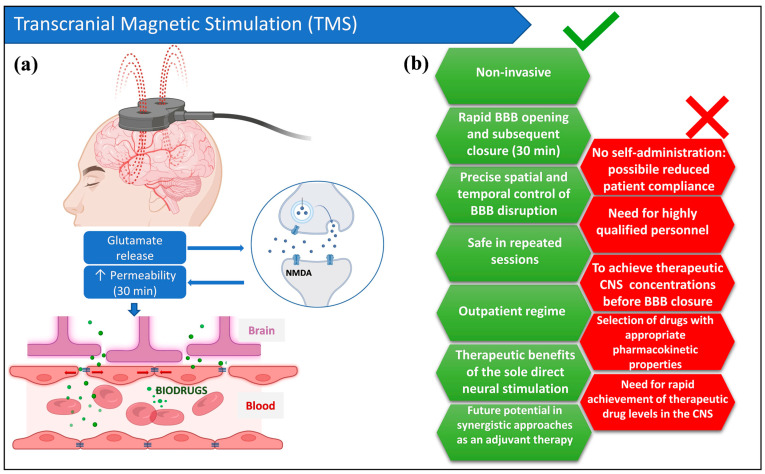
**Transcranial magnetic stimulation.** (**a**) Schematic representation of transient BBB permeabilization through the activation of NMDA receptors. The electric fields generated by TMS induce neuronal depolarization that triggers glutamate release and the consequent activation of the receptors. Rapid BBB opening and subsequent closure (30 min) represents a therapeutic window during which enhanced brain drug delivery can be achieved. (**b**) This panel shows the main strengths and limitations of the technique. Created in BioRender. Morrone, M. (2026) https://BioRender.com/9nmov1t (accessed on 30 March 2026) and modified using PowerPoint.

A systematic comparison of the key features of MRgFUS, L-PEFs and rTMS is provided in Table 1, enabling evidence-based selection of BBB opening strategies based on specific therapeutic requirements and clinical constraints. The strengths and limitations of rTMS as a CNS delivery technique are summarized in Figure 3. This figure was conceived for integrated analysis, while supporting text can be found in Section 5.

### 3.4. Intravenous Formulation for Device-Assisted Drug Delivery

In most cases, drug formulations are not intrinsically important for the mechanism of action of the device-assisted drug delivery techniques discussed thus far. Formulations are rather dictated by both the route of administration to be utilized and the physicochemical properties of the therapeutic agent, with the primary objectives of minimizing immunogenicity and maximizing bioavailability. In particular, electric field-based approaches act independently of any specific formulation to induce BBB opening, which can subsequently be exploited to enhance the delivery of pre-existing pharmacological formulations. Similarly, rTMS does not require a dedicated formulation to exert its barrier-opening mechanism, as the neuromodulatory effect is entirely mediated by the externally applied magnetic field.

In contrast, MRgFUS represents a notable exception, as the formulation plays a central role in the mechanism of action of this technique. The concurrent intravenous infusion of microbubbles, during MRgFUS application, indeed enhances the cavitation effect, thereby potentiating BBB opening and improving drug delivery to the CNS [72,74]. In this context, the physicochemical characteristics of microbubbles, including size (mean diameter and distribution), shell properties (thickness and composition), and gas core, can vary considerably across commercially available formulations, thereby influencing their response to sonication, their half-life, and their ability to modulate the BBB [137,138]. In particular, commercially available microbubbles typically range from 1 to 4.5 µm in diameter with a mean half-life of less than 5 min. Early investigations have revealed that larger bubbles (4–6 µm) increase BBB permeability, while those exceeding 8–10 µm are rapidly cleared from the circulation. The shell composition, which includes lipids, proteins, or polymers, governs the half-life and dissolution rate. Furthermore, surface functionalization with targeting ligands can be used to further enhance microbubble specificity and potency by increasing vessel wall interactions and site specificity. The gas core, typically composed of high-molecular-weight inert compounds, such as perfluoropropane (C_3_F_8_) or sulphur hexafluoride (SF_6_), is characterized by low diffusivity across the shell wall and low solubility in the surrounding aqueous medium. Of note, these are properties that collectively contribute to extend the circulatory half-life after IV injection [138].

Despite their established value, conventional microbubble formulations are subject to several inherent limitations. Their relatively large diameter confines them to the intravascular compartment, thereby restricting their targets only to biomarkers expressed within the vasculature. This decreases the potential for microbubbles to target biomarkers outside the endothelium. Furthermore, microbubbles are typically produced at low concentrations (10^6^–10^8^ bubbles/mL), and this yield compromises both shell stability and in vivo longevity. For clinical applications, microbubbles are produced by on-site “activation” protocols, which generate inherently heterogeneous bubble populations with diameters ranging from the sub-micron scale to approximately 10 µm [139]. The field has thus witnessed the emergence of nanoscale platforms to formulate nanobubbles (also referred to as sub-micron bubbles) and echogenic liposomes, which were designed not only to facilitate BBB disruption but also to extend acoustically mediated mechanical effects to the interstitial compartment [138,139,140,141].

## 4. Intranasal Delivery

The IN route of administration is emerging as a promising non-invasive, painless alternative to the oral and parenteral routes of administration to achieve therapeutic delivery into the CNS [142].

Unlike the previously described device-mediated approaches, which operate by transiently disrupting the BBB integrity, IN delivery exploits a fundamentally distinct mechanism. This approach leverages olfactory and trigeminal nerve pathways to enable drug transport from the nasal mucosa to CNS targets, effectively circumventing the BBB [143,144]. Drug formulation influences the actual mechanism mediating transport along this route that involves multiple cellular and extracellular mechanisms, including transcellular, paracellular, and extracellular pathways [144,145].

The IN route of administration has conventionally been employed for topical drug delivery in the management of localized diseases, such as allergic rhinitis, rhinoconjunctivitis, and nasal obstruction, also because of its additional benefit in exerting a humidifying effect on the nasal mucosa [2,144,146]. However, due to the extensive vascularization and enhanced permeability characteristics of the nasal mucosa, the IN route was later exploited for systemic drug delivery for a wide range of indications, ranging from pain management to hormone replacement therapy, post-menopausal osteoporosis and also emergency therapy for epileptic seizures [147,148].

IN drug delivery, as a means to achieve direct pharmacological access to the CNS, remained largely uninvestigated until 1991, when an innovative Nose-to-Brain (N2B) delivery method for targeting neurotrophic factors to the CNS was patented by Frey [149]. Following the advent of this technique, N2B delivery gained significant attention, also in consideration of its low invasiveness and related enhanced patient compliance, coupled with reduced systemic and CSF drug exposure, which potentially resulted in reduced systemic adverse effects [146,150].

Nevertheless, direct CNS penetration remains highly dependent on both the formulation features and the individual anatomical heterogeneity among subjects, with inevitable drug leakage into systemic circulation and CSF. This makes the validation of the formulation a critical requirement [144,145,146]. In addition, the rodent olfactory system exhibits significant anatomical and physiological differences when compared to humans, particularly in nasal cavity volume and morphology. These interspecies disparities can substantially hinder laboratory-to-clinic translation, as clinical outcomes in humans may deviate considerably from preclinical rodent predictions [143,151]. Such interspecies variability introduces considerable economic and temporal burdens, complicating the pathway from preclinical development to clinical applications of N2B delivery.

Beyond these translational and formulation-related challenges, additional obstacles arise at a broader level and concern the patient acceptance of intranasal administration. Consumer acceptance is influenced by several factors, including the inevitable clearance of a fraction of the administered dose through the nasopharyngeal cavity, where contact with taste receptors at the back of the tongue may elicit unpleasant sensations, such as bitterness, astringency, or saltiness. Furthermore, nasal irritation and epistaxis represent additional deterrents that may negatively impact patient adherence to the prescribed treatment regimen [152].

### IN Route for CNS Delivery of Biodrugs

The inherent capacity of the N2B delivery method to facilitate direct CNS targeting by circumventing BBB limitations and minimizing systemic exposure renders this route extremely promising for the administration of biological drugs. This delivery strategy indeed demonstrates significant potential to address critical pharmacokinetic challenges that are typical of biodrug delivery to the CNS, such as their limited membrane permeability, which prevents BBB translocation, and their enhanced systemic clearance kinetics, which synergistically contribute to inadequate drug bioavailability at target sites [143].

IN administration has proved efficient in both preclinical and clinical studies focused on CNS delivery of diverse biopharmaceutical agents, including peptides, proteins, mAbs, and gene vectors [153,154,155,156,157]. However, achieving these results requires addressing several limitations intrinsically associated with the IN route of administration, which can be further exacerbated when administrating biopharmaceuticals. Key points that need particular attention include mucociliary clearance and reduced nasal absorption, enzymatic degradation in the nasal compartment, low administrable volume and reduced reproducibility [142,158].

Given their hydrophilic properties and elevated molecular weight, biopharmaceuticals are particularly susceptible to mucus entrapment and subsequent elimination via mucociliary clearance. These events diminish the time window available for absorption, ultimately resulting in reduced brain bioavailability. Moreover, the nasal epithelium expresses multiple efflux transporters (e.g., P-glycoprotein, multidrug resistance-associated proteins) and metabolic enzymes (e.g., cytochrome P450, proteases), which create additional barriers that hinder absorption of biopharmaceuticals and thus ultimately limit the amount of drug that effectively reaches the brain [2]. In addition to these challenges, the nasal cavity imposes strict constraints on the administrable volume (0.2–0.3 mL per nostril), while repeated or prolonged exposure increases the risk of mucosal irritation and tissue damage. In this regard, biologic agents may partly offset some of these concerns, as their high potency at low doses compensates for the restricted amount delivered within the nasal cavity. Furthermore, their generally favourable biocompatibility profile may reduce the risk of mucosal damage associated with repeated administration [159].

Nonetheless, to overcome the aforementioned barriers, various formulation strategies have been developed with the final aim of improving the bioavailability in the CNS of IN administered drugs. In this regard, the intranasal route has proven suitable for accommodating a wide range of advanced formulations beyond conventional liquid suspensions and dry powders, such as nanoemulsions, nanovesicles and combination systems. In addition, a variety of excipients designed to improve drug stability and increase nasal residence time and absorption, including absorption enhancers, mucoadhesive agents, nasal enzyme inhibitors, solubilizers, and buffers, have been exploited [159]. These aspects have been extensively addressed in a previous review article published by our group and focused on the potential application of cell-penetrating peptides in intranasal formulations [2]. These strategies are further summarized in Table 2.

Herein, we will provide an overview of the results obtained with the IN delivery of different biological drugs, and we will examine the difficulties that can be encountered with this technique, thus allowing for a comparison with the previously described methods. For instance, the N2B delivery of insulin has been extensively studied for the treatment of different CNS disorders, ranging from traumatic injuries to PD, AD and cognitive impairment [173,174,175,176]. IN delivery was proven efficient in prompting the release of insulin into the CNS in both mouse and rat models. Renner et al. [177], using a solution of fluorescently labelled insulin, demonstrated that, when administered intranasally, insulin is able to travel from the olfactory epithelium along the olfactory nerve, through the cribriform plate of the ethmoid bone, to the anterior regions of the olfactory bulbs, thus effectively reaching the mouse brain. Using a more sensitive system, i.e., ^125^I-labelled insulin, Brabazon et al. [173] traced the N2B delivery of the protein in a rat model of traumatic brain injury. About 45 min after IN administration, iodinated insulin was detected not only in the olfactory bulb, though this region exhibited the highest concentration, but also in the cerebellum, brain stem, hippocampus, and cortex. Moreover, a therapeutic effect was also detected, with IN insulin enhancing both cognitive and motor performance, while concurrently attenuating hippocampal lesions and oedema, without affecting peripheral glucose homeostasis. Lochhead et al. [153] subsequently validated the ability of IN insulin to achieve widespread distribution across brain regions characterized by a high expression of insulin receptors, including the cortex, cerebellum, hippocampus, and hypothalamus, suggesting that insulin translocation involves multiple anatomical routes, extending beyond the olfactory pathway to include perineural spaces of the trigeminal nerve system.

Finally, a comparative pharmacokinetic analysis of IN versus subcutaneous insulin administration was carried out by Nedelcovych et al. [178]. This revealed that, although both routes achieved equivalent mean brain concentrations, the IN formulation displayed a 2000-fold increase in the brain-to-plasma area under the curve (AUC) ratio, with decreased plasma insulin concentrations, thereby preventing the severe hypoglycaemia observed upon subcutaneous administration.

The formulation strategies outlined above, whether applied individually or in combination, have demonstrated efficacy in the context of biopharmaceuticals, as they have also shown for other intranasal drug candidates. In the specific case of insulin, simple aqueous protein solutions have been progressively replaced by more sophisticated delivery systems, including: (i) nanoemulsion systems, such as the self-emulsifying one developed by Shah et al. [162], which significantly enhanced the absorption via the nasal route; (ii) nanovesicles, such as the chitosan–insulin–transfersomes developed by Nojoki et al. [168], which considerably improved the delivery and therapeutic effects of intranasal insulin in the brain; or (iii) combination systems, in which insulin is administered together with cell-penetrating peptides, determining enhanced nasal absorption [164].

The safety and efficacy of the IN administration of insulin has also been investigated in humans. In particular, statistically significant positive cognitive and functional outcomes, coupled with no treatment-related severe adverse events, were observed in patients with amnestic Mild Cognitive Impairment or AD, treated with IN insulin (NCT00438568) [176]. Additionally, a clinical proof-of-concept investigation (NCT02064166), involving patients diagnosed with PD or multiple system atrophy, validated the safety profile of intranasal insulin, while demonstrating amelioration of motor and cognitive manifestations [179].

N2B delivery has also been successfully evaluated for complex macromolecular therapeutics, such as mAbs. Kamei et al. [155] demonstrated that human IgG could achieve cerebral distribution in murine models, upon intranasal administration, when co-formulated with the cell-penetrating peptide L-penetratin, resulting in enhanced tissue concentrations across the olfactory bulb as well as the anterior and posterior brain regions. The calculated nose-to-brain transport percentage indicated that almost 100% of IgG was directly transported from the nose to the brain. Kamei and colleagues also evaluated the therapeutic effect of an anti-human Aβ mouse mAb (Anti-Aβ) in an AD mouse model. They found that multiple IN treatments with Anti-Aβ, co-formulated with L-penetratin, determined a significant improvement in cognitive functions by inhibiting Aβ aggregation and potentially enhancing microglial-mediated phagocytic clearance of Aβ deposits.

More complex delivery systems have been tested via the IN route to ensure targeting of monoclonal antibodies to the neural tissue, such as the mAb-loaded micelles designed by Gaikwad et al. [180] or the nanocarrier systems developed by Alves et al. [156], which comprise a lipidic core structure with surface-conjugated biopharmaceutical agents. Gaikwad and colleagues [180] developed an anti-toxic Tau conformation–specific monoclonal antibody-2 (TTCM2), which selectively binds to pathological Tau aggregates in brain sections from distinct tauopathy patients. They formulated the biodrug into lipophile micelles for IN administration to a mouse model of tauopathy. This strategy allowed the mAb to rapidly reach the brain and to exert a therapeutic action, thus ameliorating the cognitive functions of treated animals.

Alves and colleagues [156] designed and assessed two distinct delivery systems in a preclinical study based on the use of a sequential IN administration regimen, consisting of a pre-treatment followed by the therapeutic intervention. The first system was formulated with the EGFRvIII peptide (PEPvIII) (as the pre-treatment), whereas the second system was engineered to deliver the mAb Bevacizumab (as the treatment). This pharmacological approach demonstrated efficacy in a rat glioblastoma model, resulting in a reduction of 87% in the tumour size compared with the control group.

Nanocarriers have also been used for the delivery of siRNAs to the CNS through the IN route of administration. For instance, Yang et al. [157] developed a nanomicelle system, composed by the cell-penetrating peptide DP7-C surrounded by Hyaluronic Acid (HA), which was intended for IN delivery of an anti-glioma siRNA. Both its safety and efficacy were then tested in in vivo studies. The engineered HA/DP7-C nanomicelles demonstrated successful CNS penetration through the trigeminal nerve pathway, delivering the targeting siRNA to the tumour site within hours upon IN administration. The system proved effective in determining intracellular delivery of antitumoural siRNAs, thus resulting in tumour growth inhibition and prolonged survival time in tumour-bearing mice. Furthermore, safety evaluation in a rat model revealed the absence of adverse effects on the nasal mucosa or trigeminal nerve integrity. Su et al. [181] developed an exosome-based delivery system for the IN co-administration of a beta-site APP-cleaving enzyme 1 (BACE1) siRNA and berberine in a mouse model of AD. The engineered exosome delivery system demonstrated efficacy in transporting both therapeutic agents to the CNS, exhibiting enhanced bioavailability within 1 h post-intranasal administration and maintaining substantial levels at 6 h, with documented uptake by both neuronal and glial cell populations.

N2B delivery has also been assessed for cell therapy, with bone marrow stomal cells or bone marrow mesenchymal stem cells being investigated for ischemic stroke recovery [182,183]. In particular, multiple IN administrations of bone marrow stromal cells proved efficient in determining improved vascular and neural regeneration as well as functional recovery in a focal ischemic stroke model of adult mice [182].

More recently, Shen et al. [183] evaluated the therapeutic efficacy of a combinatorial therapy consisting of the intranasal co-administration of bone marrow mesenchymal stem cells with Insulin-like Growth Factor-1 (IGF-1), a potent anti-apoptotic agent. This dual therapeutic strategy enhanced angiogenesis and neurogenesis around the ischemic region in a focal cerebral ischemia model, culminating in significant neurological function improvement.

Recent initiatives have focused on clinical translations of this cellular therapeutic approach. A multicentre clinical trial, namely Neurologic Bone Marrow Derived Stem Cell Treatment Study (NEST, NCT02795052), is presently recruiting participants to investigate the combined administration of systemic infusion and intranasal delivery of bone marrow stem cells for the treatment of functional damage to the central or peripheral nervous system. However, preliminary findings have not yet been disclosed [184].

Collectively, the intranasal route of administration represents a valuable alternative to the parenteral delivery of various CNS therapeutics. Due to its non-invasive nature and potential for self-administration, this approach offers significant advantages in ensuring improved patient compliance with treatment regimens. Nevertheless, a major challenge remains, namely, achieving adequate drug concentrations at the target site with current administration devices, due to the inaccuracy in the administration techniques [2,185]. This inaccuracy stems from the interplay of multiple factors, including (1) patient-related characteristics, such as a deviated nasal septum; (2) formulation properties, such as low viscosity; (3) device-related parameters, such as particle size and plume geometry; and (4) administration errors, such as incorrect head positioning. All of this may ultimately result in product drip-out and incomplete dosing at the absorption site [152]. Partial resolution of these challenges has been achieved through the development of advanced delivery devices, such as Intravail™, which facilitate more precise and reproducible intranasal drug delivery when compared to conventional nasal spray bottles [186].

**Figure 4:**
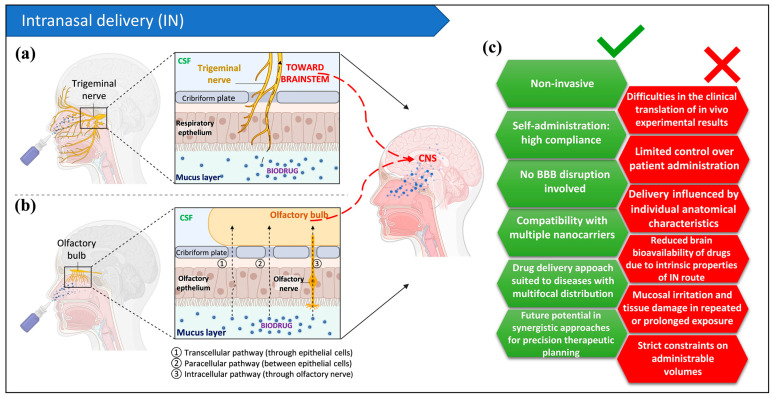
**Intranasal delivery.** Schematic representation of the two primary pathways allowing for the BBB bypass upon intranasal dosing: (**a**) the trigeminal nerve pathway and (**b**) the olfactory pathway, both of which establish connections with the CNS through multiple mechanisms (*lower panel*, 1–3). (**c**) This panel shows the main strengths and limitations of the technique. Created in BioRender. Morrone, M. (2026) https://BioRender.com/xrz4cl4 (accessed on 30 March 2026) and modified using PowerPoint.

A brief analysis of the strengths and limitations of this delivery route is presented in Figure 4, while a more complete discussion can be found in Section 5.

## 5. IN Delivery vs. Device-Assisted Drug Delivery in Different Neurological Disorders

The risk–benefit assessment constitutes the foundation of all phases of drug development, from initial molecular design and route of administration selection to regulatory agency approval. Determining acceptable risk levels for a given treatment in relation to its therapeutic benefits presents considerable complexity [187].

Selecting a specific delivery strategy for agents targeting the CNS represents an aspect intimately linked to the risk–benefit analysis. Comprehensive evaluation requires consideration of the pathological condition, its aggressiveness, the importance of delivery precision, and the maintenance of an appropriate degree of invasiveness [67].

A factor that researchers tend to underestimate involves understanding patient treatment priorities. More specifically, patient consultation data demonstrate that a major concern, beyond adverse event mitigation, focuses on maintaining functional independence to perform habitual activities throughout the treatment course [188].

In this regard, a major distinction among the aforementioned techniques, which may significantly impact the ability of patients to maintain a normal life, is the option for self-administration that is exclusively available via the IN route [2].

All device-assisted drug delivery techniques must indeed be performed in a medical facility. With the exception of IRE, which requires execution in a surgical setting under general anaesthesia for electrode placement in the skull, all other methods can be conducted on an outpatient basis, with medical professionals supervising the concurrent IV drug infusion and, when needed, providing image guidance [81,189,190]. The fact that the administration requires the use of sophisticated equipment, such as MRI scanners or infusion pumps, in addition to trained personnel, generates significant expenses for the healthcare facility and the national healthcare system [191]. Nevertheless, a study designed to analyse the cost-effectiveness of MRgFUS for the treatment of patients affected by essential tremor led to the conclusion that the procedure was favourable from the perspective of the National Health System of England [192]. On the other hand, self-administration can have pitfalls, since it is unfeasible to monitor proper execution, and thus it can expose the patient to dosing fluctuations, especially when medical devices lack a user-friendly design [185].

A further significant distinction resides in the intrinsic risks of the mechanisms of action of these approaches. Whereas IN administration operates by circumventing the BBB, device-assisted techniques cause BBB disruption that, in addition to being time-restricted, may expose the CNS to neurotoxic substances [9].

In the case of device-assisted techniques, different methodologies determine BBB opening with varying temporal dynamics, with TMS producing barrier disruption lasting approximately 30 min, while MRgFUS maintains the opening for around 24 h and IRE for 72 h [109,121,130,193]. From this perspective, IRE, in addition to requiring invasive electrode insertion procedures, presents the highest risk profile due to its extended BBB disruption period.

On the other hand, IN delivery may induce, depending on the drug and repeated administration, mucosal toxicity sufficient to compromise patient tolerability and alter drug absorption, a concern of particular relevance in the management of chronic conditions requiring recurrent dosing [159].

A further substantial difference between device-assisted and IN delivery that must be considered in therapeutic strategy selection is the precision of drug delivery, with IN administration resulting in drug diffusion across multiple CNS regions, whereas device-assisted techniques achieve more precise localization [153]. When evaluating this parameter, the focality of the underlying pathological condition to be treated must be, therefore, taken into account [67,76].

An additional aspect to consider when selecting CNS delivery techniques for drugs targeting neurological pathologies is the therapeutic dosing frequency and treatment duration requirements. In this regard, all non-invasive approaches described in this review have proven to be safe for repeated administration across sequential treatment sessions, which provides greater treatment flexibility [119,176,190,194].

Finally, an interesting aspect is the potential for the described techniques to be combined, e.g., to enhance the delivery of therapy. In this regard, the possibility of utilizing TMS to enhance the delivery of IN-administered magnetic nanoparticles to target sites has recently been investigated in vivo [195]. This application diverges from the role of TMS as a modality for enhancing delivery across the BBB and exploits it as a mechanism for drug attraction to the target site, while simultaneously opening avenues for technique combination approaches. One potential future approach could be, for example, that of combining device-assisted sessions with home-based maintenance therapy administered by IN delivery.

Beyond clinical considerations, the commercial landscape of therapeutic innovation is significantly shaped by the developmental costs intrinsic to each competing technology. Both device-assisted and intranasal delivery approaches involve the combined use of a pharmacological agent and a dedicated device, each of which requires independent design and development to meet specific therapeutic requirements. This complexity is further compounded when the pharmacological component belongs to the biological drug class, whose development process is precisely what drives their elevated cost, averaging between $10,000 and $30,000 per patient per year, and exceeding $500,000 for the most expensive agents in this class [196,197]. Given that the development of a single biological agent may represent a corporate investment of up to $1.8 billion, minimising developmental risks and complexity at every stage becomes a critical strategic priority [197]. In this respect, device-assisted approaches present a potentially advantageous profile, as outlined in Section 3. Such platforms may accommodate already-approved formulations with targeted modifications, such as the incorporation of microbubbles in the context of MRgFUS, thereby reducing formulation-related costs. Intranasal delivery, by contrast, poses a more demanding developmental challenge, particularly for biological compounds, where formulation represents the primary and most complex hurdle. This challenge is further amplified by the limited translational fidelity of preclinical models, thus introducing a significant economic liability. Discrepancies between preclinical and clinical outcomes may indeed necessitate costly redevelopment cycles, further prolonging and financially burdening an already complex developmental pipeline [143,151].

In the following sections, we will analyse the potential of individual approaches in the treatment of brain tumours and neurodegenerative diseases.

### 5.1. GBM

As previously stated, GBM is the most common and lethal malignant primary brain tumour in adults. Current standard of care is represented by a quite complex approach, combining initial tumour resection with subsequent concomitant radiotherapy and chemotherapy with the alkylating agent TMZ. Oral TMZ, which was shown to extend average GBM survival by several months when compared to radiation alone, is known to penetrate the BBB, but the efficiency is low, with an only 0.2 brain:plasma ratio [198,199]. The outcomes of this conventional approach are not satisfactory [28]. Indeed, the Overall Survival (OS) for GBM is set at 68% at 1 year, with average OS under two years (12.8–14.6 months). Moreover, GBM is characterized by a markedly high recurrence rate and a Progression-Free Survival (PFS) of merely 7–7.5 months after a primary treatment [200,201]. Given the limited number of effective standard therapeutic options, clinical trial enrolment should be pursued whenever feasible [28,202].

Considering the aggressive nature of the disease and the invasiveness of conventional treatment modalities, the implementation with some of the higher-risk techniques presented in this review, such as ultrasound-mediated thermoablation or IRE-mediated ablation, may constitute an acceptable therapeutic strategy.

MRI-guided High-Intensity Focused Ultrasound (HIFU) demonstrated early therapeutic potential in thermal ablation of brain neoplasms. However, this technique was ultimately abandoned due to the limitations in the achievable treatment field and the extensive time requirements for substantial tumour volume reduction [203,204]. In addition, H-FIRE-mediated ablation is under clinical investigation for diverse malignancies outside the CNS, such as prostate cancer (NCT03838432; NCT05345444) and pancreatic cancer (NCT01939665; NCT02791503); but, to date, no clinical applications in GBM have been documented [205,206,207,208,209].

Conversely, clinical investigations have been undertaken for less aggressive approaches, including low-intensity MRgFUS and IN drug delivery applications in an attempt to ameliorate the BBB permeability of TMZ.

The first-in-human proof-of-concept study (NCT02343991) for non-invasive transcranial MRgFUS, combined with chemotherapy (liposomal doxorubicin and TMZ), demonstrated the safety and tolerability of image-guided BBB disruption, with a lack of adverse clinical or radiological events related to the procedure in a reduced number of individuals. Unfortunately, analysis of drug distribution within the tumour mass generated inconclusive outcomes in this study [210].

Importantly, the results of the NCT03712293 trial further corroborated the safety of low-intensity energy MRgFUS, as a means to disrupt the BBB along the periphery of the tumour resection cavity in GBM patients undergoing TMZ therapy after surgical excision [194].

From an efficacy standpoint, one-year follow-up data demonstrate highly encouraging outcomes and suggest potential therapeutic advantages in pairing MRgFUS with TMZ therapy. Trial participants achieved a 100% OS rate at 12 months and a median PFS of 15 months, surpassing standard disease benchmarks [211].

Promising preliminary results have also been documented with the combination of microbubble-enhanced MRgFUS and Bevacizumab, a vascular normalizing agent that extends PFS and helps with symptom management in patients with recurrent GBM (NCT04446416). This therapeutic strategy was found to be safe and clinically feasible [30,212].

N2B delivery strategies are also currently under investigation for GBM treatment. For instance, the phase I pilot study NCT04091503 was designed to evaluate the safety, tolerability, and potential efficacy of IN TMZ administration as a single agent in GBM patients, underscoring the significant clinical interest in this therapeutic approach. Unfortunately, although this study appears to be completed, no results are currently available [213]. Moreover, a phase I/IIa clinical trial (NCT02704858) is currently ongoing to assess the safety, pharmacokinetics and efficacy of another agent, namely NEO100 (perillyl alcohol), in a self-administration repeated dose regimen, in patients with Grade IV glioma. Phase I results indicate that IN NEO100 treatment is well tolerated and correlated with improved OS in comparison to historical controls, suggesting the clinical potential of this novel intranasal delivery system in recurrent GBM therapy [214].

As per the other non-invasive techniques described in this review, there is currently very limited or no clinical evidence regarding the efficacy of L-PEFs and TMS for the treatment of GBM, despite promising preclinical assessments. Yet, an initial pilot trial in humans (NCT02474966), involving glioma patients, demonstrated the safety of an rTMS approach for enhancing CNS delivery of systemically administered tracers [132].

In conclusion, despite considerable efforts being made toward the validation of novel technologies and delivery methods for GBM treatment, these therapeutic approaches remain in early developmental phases. Treating GBM presents significant challenges as it involves: (a) managing an extremely aggressive malignancy requiring surgical resection and margin clearance; (b) frequent development of resistance to first-line agents; and (c) high recurrence rates due to migrated cells beyond tumour margins [28,30,31].

The use of device-assisted strategies for CNS delivery may help delivering drugs to tumour margins yet may prove inadequate for targeting individual cells that have migrated from the margins [8]. Therefore, a combined approach with a non-invasive technique that limits systemic exposure, and consequently side effects, such as the IN route of administration, may help reach even dispersed individual cells. Nonetheless, one issue that remains to be addressed is the development of resistance to TMZ. Thus, it becomes clear that alternative therapeutic options must be identified.

### 5.2. Neurodegenerative Diseases

Although considerable advances have been made in characterizing the molecular pathogenesis of several neurodegenerative diseases, these disorders remain invariably progressive, disabling, and ultimately fatal conditions. While numerous strategies have demonstrated neuroprotective properties in preclinical in vitro and in vivo models of neurodegeneration, the vast majority of neurodegenerative conditions lacks clinically meaningful therapeutic options [215]. For instance, currently available therapeutic interventions for ALS achieve modest attenuation of motor functional deterioration but fail to induce genuine disease regression or pathological reversal. Rilutek (riluzole), a neuroprotective small molecule approved by both the EMA and FDA, demonstrates limited therapeutic efficacy in ALS, primarily manifesting as a 2–3-month extension in tracheostomy-free survival. Similarly, Radicava ORS (edaravone), an oral antioxidant compound approved exclusively by the FDA, produces a reduced rate of decline in validated clinical measures of daily functional capacity [216,217,218]. The first targeted therapy for ALS, Qalsody (Tofersen), consisting in an antisense oligonucleotide indicated for the treatment of the familiar form of ALS linked to *SOD1* mutations, demonstrates efficacy on surrogate endpoints likely to predict clinical benefits in the pivotal phase 3 authorization trial. However, it failed to achieve statistically significant improvements in primary clinical endpoints [219,220].

In the case of MS, multiple DMTs have been approved to date for RRMS, including IFNβ-1a (Avonex), IFNβ-1b (Betaseron or Extavia), glatiramer acetate (Copaxone), Natalizumab (Tysabri), fingolimod (Gilenya), and dimethyl fumarate (Tecfi). In spite of demonstrated efficacy in reducing the inflammatory reaction, relapse frequency, and disability progression, prolonged treatment remains complicated by safety considerations, patient-specific immunological variability, and adherence challenges [38,40,41].

Regarding the therapeutic approaches for AD, conventional pharmacological intervention based on anticholinesterase inhibitors (donepezil, galantamine, and rivastigmine) and anti-glutamatergic compounds (memantine) have traditionally focused on symptom management rather than disease modification [44,45]. Only in recent years, the disease-modifying monoclonal antibodies Aducanumab and Lecanemab, which target Aβ plaques, were made available on the market [46,47]. However, according to the United States Prescribing Information, treatment with Lecanemab is indicated only in cases with Mild Cognitive Impairment or a mild dementia stage and a confirmed presence of Aβ pathology, thus restricting the perspective of eligible patients. No safety or efficacy data are currently available for earlier or later stages. On the other hand, the approval of Aducanumab by the FDA generated considerable controversy, principally due to discordant findings from two methodologically identical phase 3 trials (NCT02477800 and NCT02484547) as well as insufficient evidence establishing a correlation between cerebral Aβ clearance and clinical benefits. This led to EMA’s refusal of grant marketing authorization [47]. Essentially, the identification of therapies with significant disease-modifying properties remains a critical unmet medical need for these neurodegenerative pathologies, with insufficient BBB penetration of therapeutically promising compounds constituting a major impediment to effective clinical translation [215]. In this regard, the alternative delivery approaches presented in this review could prove beneficial.

Following promising preclinical results, MRgFUS is beginning to be translated into clinical applications for the treatment of neurodegenerative diseases. Given its experimental nature, preliminary clinical investigations are being performed without concurrent disease-modifying drug administration, focusing exclusively on safety characterizations of the technique. For instance, the first-in-human trial of MRgFUS in ALS (NCT03321487) was focused solely on assessing the feasibility and safety of primary motor cortex BBB opening using an MRgFUS device. In this trial, the intravenous ultrasound contrast agent gadolinium was employed to evaluate the capacity of MRgFUS to facilitate targeted delivery to the motor cortex, with no expectation of therapeutic benefits due to the lack of concurrent drug administration. Millimetric accuracy in targeting the motor cortex was demonstrated, as well as the lack of SAEs, haemorrhage or persistent lesions, indicating high tolerability [193].

Similar results were achieved in five patients with early to moderate AD in a phase I safety trial (NCT02986932). While the hippocampus might be considered more clinically relevant, the frontal cortex was chosen as the target in this trial, in order to mitigate the risk of oedema and haemorrhage linked to the hippocampal deep localization. By using gadolinium extravasation again to assess BBB opening, this study demonstrated both feasibility and safety of MRgFUS in AD patients. Notably, during this trial, the procedure was repeated twice at one-month intervals in compliant patients. The outcome of this procedure demonstrated the safety of repeated sessions, which may be needed for the treatment of these disorders [221].

Preliminary results from a subsequent ongoing trial (NCT03671889) confirmed the safety of MRgFUS-mediated BBB opening in AD patients using the hippocampal region as the target. During this trial a total of 17 interventions were performed across six patients demonstrating acceptable treatment tolerability with no reported adverse events or deterioration in cognitive or neurological functions [222]. Safety, feasibility and reversibility of repeated MRgFUS-mediated BBB disruption sessions were also demonstrated in patients affected by PD in an ongoing phase I clinical trial (NCT03608553). Reversible BBB opening in the parieto-occipito-temporal junction and posterior putamen was reported without SAEs, such as haemorrhage or oedema [75,223]. Clinical development has progressed further, with an approved pharmaceutical agent, named Cerezyme^®^, being investigated in a phase I pilot study in combination with MRgFUS as a potential DMT for Parkinson’s Disease (NCT04370665). Study outcomes support both the safety profile and clinical feasibility of MRgFUS in combination with Cerezyme^®^ for PD treatment [81].

Concurrently, the therapeutic potential of rTMS is gaining considerable attention in the field of neurologic diseases. Although rTMS has become an established therapeutic intervention for psychiatric disorders, including major depressive disorder and obsessive-compulsive disorder, recent interest has increasingly shifted toward diverse neurological disorders, such as AD and PD [224]. While a few clinical trials have been undertaken, none has investigated the potential of this technique to enhance CNS delivery of DMTs through BBB disruption in this field. Nevertheless, rTMS merits consideration for neurodegenerative disease treatment based on the therapeutic benefits achieved via direct neural stimulation. For instance, through a meta-analysis encompassing 20 different studies, Chou et al. demonstrated that rTMS intervention has a significant effect on motor function capacity in patients affected by PD, achieving what it is classified as a moderate clinically important difference [225].

Furthermore, a subsequent meta-analysis of randomized controlled trials (RCTs) conducted by Pagali et al. demonstrated significant enhancement of both global and specific cognitive functions in patients with Mild Cognitive Impairment and AD following rTMS treatment. The analysis further established the favourable safety and tolerability profile of rTMS in these clinical populations [226].

Taken together, these results indicate the potential of rTMS as a complementary therapeutic intervention in the management of neurodegenerative disorders. Future investigations would benefit from examining its synergistic efficacy when combined with DMT delivery.

Beyond being in a preliminary phase of development, all prospective therapeutic interventions analysed thus far eventually require patients to repeatedly attend outpatient facilities over a long term to receive treatments. Moreover, MRgFUS necessitates head shaving prior to treatment, which may adversely impact patient compliance [215]. In addition, both aforementioned approaches require precise target identification, rendering them effective when lesions are characteristically confined to discrete anatomical regions, such as in PD. However, in diseases like MS, characterized by multifocal lesion distribution, a more widespread therapeutic approach is necessary, which may be achieved through IN delivery [67].

Clinical translation of IN therapeutic delivery for neurodegenerative diseases has progressed to a more advanced developmental stage than device-assisted methodologies. Specifically, substantial clinical research, encompassing phase II studies, has been conducted in both PD and AD, focusing on intranasal protein administration protocols [227]. For instance, among clinical trials investigating IN drug delivery in PD, approximately half of the studies involve therapeutic protocols cantered on insulin and/or glutathione as primary therapeutic agents [227]. A phase II proof-of-concept trial (NCT02064166), involving 16 participants with clinically diagnosed PD or multiple system atrophy, demonstrated the feasibility and safety of IN insulin for the treatment of PD, while additionally documenting enhanced motor and cognitive outcomes in the active treatment arm compared to pre-treatment baselines and placebo controls [179]. Similarly, two phase I studies (NCT02324426 and NCT01398748) have demonstrated the safety of IN glutathione for the treatment of PD [228,229]. A subsequent phase IIb (NCT02424708) study was therefore conducted to evaluate treatment effectiveness, although the results did not reach definitive conclusions [230].

Likewise, N2B delivery systems have been explored as options for therapeutic interventions for AD management utilizing insulin-based protocols. In particular a phase II trial (NCT00438568) and a phase II/III trial (NCT01767909) demonstrated feasibility and safety of the approach, with no SAEs detected. However, while the NCT00438568 trial provided evidence supporting the cognitive benefits of intranasal insulin intervention, the subsequent trial, NCT01767909, failed to replicate these findings, demonstrating no measurable cognitive or functional improvements over the 12-month treatment duration [176,185].

Confirming the constant interest in IN delivery, two compelling trials are currently underway, investigating this route of administration for cell-based therapies (NCT03724136 and NCT02795052). Yet, preliminary findings have not yet been disclosed [184,231].

The IN route of delivery is also under investigation for the treatment of MS. Interestingly, several preclinical studies have explored N2B delivery as a non-invasive strategy for targeting FDA-approved MS therapies to the CNS. In this regard, remarkable results were achieved with the IN administration of the anti-inflammatory cytokine IFNβ-1b, a first-line systemic therapy for MS patients, with both free and nanoparticle formulations, allowing for the achievement of superior brain penetration and drug concentrations compared to the conventional IV route [232,233]. Regarding clinical applications, a phase Ib/II clinical (NCT02988401) study primarily investigating the feasibility and safety, and secondarily the efficacy on cognitive and memory improvement of multiple IN administrations of insulin in MS patients has recently been completed. The approach was demonstrated to be well-tolerated and safe, with only a few cases of SAEs unrelated to the drug and equally distributed across the study treatment groups. However, IN insulin was not superior to a placebo in any of the clinical outcome measures assessed [234].

Collectively, these findings suggest that, although intranasal delivery has progressed to more advanced developmental phases where therapeutic efficacy is empirically evaluated, significant barriers remain in translating preclinical success to clinical outcomes. This may be due either to the fact that efficient DMTs have not yet been identified or to the insufficient delivery to the site of action, possibly because of the poor control over the correctness of the administration method, as suggested by Craft et al. [185]. It is also reasonable to postulate that IN delivery can provide a broad drug distribution, while device-assisted techniques might target specific symptomatic lesions. This suggests that two approaches may be complementary in the treatment of MS. In summary, the critical factors to evaluate when determining drug delivery approaches for neurodegenerative pathologies include disease focality and patient compliance, particularly considering the chronic nature of these treatments, which requires sustained therapeutic engagement. Regarding the first aspect, a treatment based on the use of the intranasal route, or a combined treatment that integrates it, may be advantageous for pathologies presenting multifocality. Concerning the second aspect, the potential for self-administration, although still requiring refinement, may be superior to treatments requiring outpatient care regimens.

While beyond the scope of this discussion, it would be crucial to consider in neurodegenerative therapeutic design that these diseases may manifest effects in other tissues, such as, for example, muscle tissue. This presupposes a broader therapeutic design that potentially combines systemic and targeted adjuvant therapies with ad hoc rehabilitation plans.

## 6. Concluding Remarks

This review has presented diverse CNS delivery methodologies at various developmental stages. Device-assisted techniques, encompassing MRgFUS, electroporation (particularly L-PEFs), and rTMS, employ external mechanical, electrical, or alternative stimuli to induce BBB permeabilization, thereby facilitating targeted therapeutic delivery to specific CNS regions. As discussed herein, these approaches, while maintaining non-invasive or minimally invasive profiles, contrast with strategies employing unconventional administration pathways, such as IN delivery. The latter is indeed designed to entirely circumvent the BBB without subjecting patients to risks correlated to uncontrolled barrier disruption. All methodologies, regardless of their developmental phase, have demonstrated efficacy in delivering to CNS targets diverse pharmaceutical formulations, particularly biotechnological therapeutics characterized by elevated molecular weights. Certain device-assisted modalities, notably rTMS, exhibit additional value as adjunctive therapies due to their inherent therapeutic benefits. The principal clinical differences among the discussed approaches, aside from their intrinsic delivery mechanisms, encompass administration regimens and target specificity. For example, outpatient protocols for device-assisted methodologies contrast with autonomous patient administration in the case of IN delivery. Furthermore, device-assisted techniques offer focal drug delivery to specific brain regions when compared to the more diffuse distribution achieved through the IN route.

From a commercial development standpoint, the primary challenges diverge between the two strategies. For device-assisted approaches, the principal bottleneck lies in technological advancement of the device itself—such as, for example, simplifying the coil design in rTMS, while preserving therapeutic performance. For intranasal delivery, the critical hurdles are twofold: the formulation of biological agents capable of crossing the nasal mucosa and the limited translational validity of preclinical findings, which can be ascribed to the structural discrepancies between rodent and human nasal anatomy.

From a regulatory standpoint, it is worth noting that while both approaches involve the combined use of a medicinal product and a medical device—and are therefore subject to both EU MDR 745/2017 and Directive 2001/83/EC in the EU and to the FDA’s regulatory frameworks for medical devices and drugs in the U.S.—only those intranasal delivery systems in which the medical device constitutes an integral component of the pharmaceutical product may be classified as a drug–device combination system under Article 1(8) of the MDR in EU or 21 CFR Part 3—Combination Products in the U.S. In such cases, the primary mode of action determines that the regulatory pathway follows the Directive, whilst the manufacturer is nonetheless required to incorporate device-specific information within the Common Technical Document. This additional regulatory burden, associated with the combined product classification, may consequently extend the time to market of the pharmacological substance, representing a further commercial consideration in the developmental strategy of intranasal delivery platforms.

Finally, the complexity of the disorders taken under consideration (i.e., GBM and neurodegenerative diseases), coupled with the heterogeneous and preliminary developmental phases of the delivery methodologies as well as the challenge of establishing effective DMTs, renders it difficult to definitively establish the superiority of one approach over the other in each specific scenario.

A prospective approach emphasized by this work involves the development of integrated protocols, where multiple delivery techniques can be combined to address the distinct therapeutic requirements presented by specific pathological conditions, also considering the growing emphasis on personalized medicine.

## Figures and Tables

**Table 1:** **Quantitative and qualitative comparison of device-assisted BBB opening techniques for enhanced CNS drug delivery.** This table assesses the three major non-invasive BBB disruption technologies across five critical parameters that determine clinical applicability and therapeutic efficacy. In particular, BBB Opening Duration quantifies the temporal window of barrier permeabilization, revealing a spectrum from ultra-brief (<30 min for rTMS, requiring concurrent drug administration) to intermediate (up to 24 h for MRgFUS with microbubbles). Spatial Precision evaluates the capacity to achieve targeted, localized BBB disruption, with MRgFUS demonstrating the highest precision through MRI-guided millimetre-scale accuracy, whereas L-PEFs exhibit spatial heterogeneity. Clinical Readiness stratifies techniques along the translational pipeline from proof-of-concept through clinical validation, with MRgFUS representing the most clinically advanced approach. Patient Burden assesses invasiveness and procedural complexity, while Technical Challenge emphasizes the most challenging design consideration.

Technique	BBB Opening Duration	Spatial Precision	Clinical Readiness	Patient Burden	Technical Challenge
MRgFUS	Transient disruption: up to 24 h (with microbubbles).	High spatial precision, MRI-guided targeting, and millimetric accuracy.	Clinical stage: - Open-label phase I trial for recombinant-protein delivery (NCT04370665). - Open-label phase I trial for mAb delivery (NCT03714243).	Non-invasive, and requires microbubble IV injection and MRI session time. Multiple sessions possible.	Ultrasound beam aberrations caused by the skull.
L-PEFs	Temporal limit not fully characterized.	Uneven spatial distribution, determining spatial heterogeneity in drug delivery. BBB opening extends beyond targeted area.	Preclinical stage.	Non-invasive, and requires IV drug administration. Limited clinical data on session requirements.	Only proof-of-concept prototypes have been developed, with no safety characterization or regulatory pathway established for human use.
rTMS	Very brief opening window <30 min. Rapid BBB closure.	Region-specific modulation. Quantitative data not yet available.	Early clinical stage, with a pilot trial in humans (NCT02474966).	Non-invasive, and requires IV drug administration. Safe for repeated sessions.	Simplified coil design, while maintaining performance.

**Table 2:** **Formulation strategies to overcome the limited brain bioavailability of intranasally administered drugs.** The mechanism by which each approach enhances CNS exposure is described. Strategies are grouped according to the primary pharmacokinetic barrier they tackle.

Proposed Strategies to Overcome Reduced Brain Bioavailability		Mechanism	Refs.
To enhance drug solubility in the nasal cavity.	Encapsulation in cyclodextrins	Upon inclusion of complex formation, the poorly water-soluble drug is entrapped within the hydrophobic core, while the outer hydrophilic shell ensures solubilization of the complex and facilitates its penetration across the mucus layer.	[160,161]
	Microemulsion and nanoemulsion formulation	As stable colloidal systems comprise an aqueous phase and an oil phase, micro- and nanoemulsions enable the formulation of hydrophobic small molecules or even proteins and/or peptides that are either water-insoluble or prone to precipitation at physiological pH.	[162]
	Encapsulation into liposomes	As phospholipid bilayer vesicles comprise an aqueous core and a hydrophobic membrane, liposomes serve as versatile carriers for both hydrophilic and lipophilic drugs. They enhance drug solubility, while facilitating mucosal permeation.	[159,163]
To enhance drug permeation across the nasal epithelium.	Functionalization/co-administration with cell-penetrating peptides	Cell-penetrating peptides facilitate drug transport across the nasal epithelium by directly penetrating the lipid bilayer of epithelial cells, enabling transcellular translocation independent of active transport mechanisms.	[164,165]
To reduce the clearance, prolonging the residence time of the formulation at the delivery site.	Mucoadhesive and viscosity-enhancing agents	Mucoadhesive agents enhance drug absorption by prolonging residence time in the nasal cavity through adhesive interactions with the nasal mucosa, thereby reducing mucociliary clearance. Additionally, the mucoadhesive polymer chitosan can electrostatically interact with the negatively charged surface of nasal epithelial cells, further extending nasal retention and promoting the uptake of drug-loaded nanoparticles.	[159,166,167,168]
	Reversible and irreversible ciliostatic and ciliotoxic drugs	There is a long list of both reversible and irreversible ciliostatic and ciliotoxic drugs. Ciliostatic agents impair ciliary motility, thereby reducing mucus clearance, whereas ciliotoxic compounds exert a more detrimental effect by directly damaging the structural integrity of the cilia or the underlying epithelium.	[169]
	Formulation in hydrogels	Hydrogels are three-dimensional hydrophilic polymer networks capable of retaining large amounts of water and biological fluids. They represent a versatile platform for drug loading, offering extended mucosal retention time, enhanced intranasal uptake, and protection against chemical and enzymatic degradation within the nasal passages.	[166]
	Transporter inhibitors	Efflux transporters, such as P-glycoprotein, limit brain uptake of IN-administered therapeutics. Transporter inhibitors (e.g., rifampicin) effectively counteract this mechanism, resulting in greater CNS drug delivery.	[170,171]
	Vasoconstrictors	Vasoconstrictors minimize drug clearance into the systemic circulation, thereby enhancing delivery through the N2B pathway.	[172]
To reduce degradation by enzymes and proteases in the nasal cavity.	Enzyme inhibitors	Nasal enzyme inhibitors are employed to minimize the drug degradation occurring within the nasal cavity and may be administered either prior to or concomitantly with the drug of interest.	[159,169]

## Data Availability

No new data were created or analyzed in this study. Data sharing is not applicable to this article.

## Abbreviations

The following abbreviations are used in this manuscript:

AAV	Adeno-Associated Virus
AD	Alzheimer’s Disease
AJ	Adherens Junction
ALS	Amyotrophic Lateral Sclerosis
AUC	Area Under the Curve
Aβ	Amyloid β
BACE1	Beta-Site APP-Cleaving Enzyme 1
BBB	Blood–Brain Barrier
BCSFB	Blood–Cerebrospinal Fluid Barrier
B-SWE	Burst Sine Wave Electroporation
BTB	Blood–Tumour Barrier
CED	Convection-Enhanced Delivery
CIS	Clinically Isolated Syndrome
CNS	Central Nervous System
CP	Choroid Plexus
CSF	Cerebrospinal Fluid
DMT	Disease-Modifying Treatment
EC	Endothelial Cell
ECT	Electrochemotherapy
EMA	European Medicines Agency
FDA	Food and Drug Administration
FUS	Focused Ultrasound
GBM	Glioblastoma Multiforme
GCase	Glucocerebrosidase
GCPII	Glutamate carboxypeptidase II
HA	Hyaluronic Acid
H-FIRE	High-Frequency Irreversible Electroporation
HIFU	High-Intensity Focused Ultrasound
IGF-1	Insulin-like Growth Factor-1
IGF-Trap	Insulin-like Growth Factor Trap
IN	Intranasal
IRE	Irreversible Electroporation
IV	Intravenous
LITT	Laser Interstitial Thermal Therapy
L-PEFs	Low Intensity PEFs
mAb	Monoclonal Antibody
MDC	Microbubble-Drug Conjugate
MRgFUS	Magnetic Resonance-Guided Focused Ultrasound
MRI	Magnetic Resonance Imaging
MS	Multiple Sclerosis
N2B	Nose to Brain
NaFlu	Sodium Fluorescein
NVU	Neurovascular Unit
OS	Overall Survival
PD	Parkinson’s Disease
PEFs	Pulsed Electric Fields
PEPvIII	EGFRvIII Peptide
PFS	Progression-Free Survival
PPMS	Primary Progressive MS
RCT	Randomized Controlled Trial
RE	Reversable Electroporation
RRMS	Relapsing–Remitting MS
rTMS	Repetitive Transcranial Magnetic Stimulation
SAE	Serious Adverse Event
SPMS	Secondary Progressive MS
TJ	Tight Junction
TMS	Transcranial Magnetic Stimulation
TMZ	Temozolomide
TTCM2	Toxic Tau Conformation–Specific Monoclonal Antibody-2
